# {μ-6,6′-Dimeth­oxy-2,2′-[cyclo­hexane-1,2-diylbis(nitrilo­methyl­idyne)]diphenolato}trinitratocopper(II)lutetium(III)

**DOI:** 10.1107/S1600536810048245

**Published:** 2010-11-27

**Authors:** Yan Bao, Guang-Ming Li, Fan Yang, Peng-Fei Yan, Peng Chen

**Affiliations:** a School of Chemistry and Materials Science, Heilongjiang University, Harbin 150080, People’s Republic of China

## Abstract

In the title dinuclear Cu^II^–Lu^III^ salen-type complex, [CuLu(C_22_H_24_N_2_O_4_)(NO_3_)_3_], with the ligand 6,6′-dimeth­oxy-2,2′-[cyclo­hexane-1,2-diylbis(nitrilo­methyl­idyne)]diphenolate, the irregular nine-coordinate Lu^III^ coordination sphere comprises four O atoms from the ligand and five O atoms from three nitrate groups, two bidentate and one monodentate [Lu—O = 2.230 (3)–2.621 (4) Å]. The slightly distorted square-planar four-coordinate Cu^II^ atom comprises two imine N atoms [Cu—N = 1.903 (4) and 1.912 (4) Å] and two phenolate O atoms from the ligand mol­ecule [Cu—O = 1.897 (3) and 1.906 (3) Å]. All atoms of the cyclo­hexane ring of the ligand mol­ecule are disordered over two sets of sites with equal occupancy.

## Related literature

For the synthesis of the ligand, see: Aslantaş *et al.* (2007 ▶); Mohamed *et al.* (2003 ▶). For similar copper–lanthanide complexes of this salen-like ligand, see: Bao *et al.* (2010 ▶); Koner *et al.* (2005 ▶); Sui *et al.* (2006 ▶); Costes *et al.* (2008 ▶); Sun *et al.* (2009 ▶).
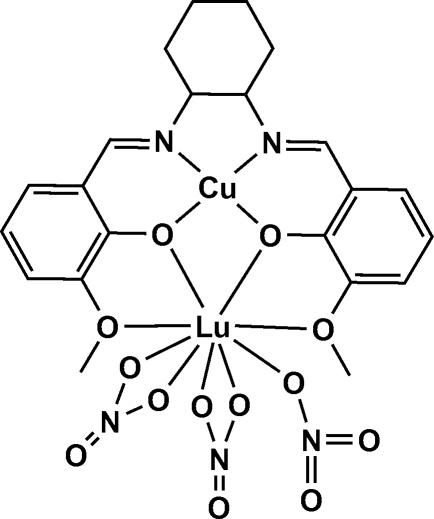


## Experimental

### 

#### Crystal data

[CuLu(C_22_H_24_N_2_O_4_)(NO_3_)_3_]
*M* *_r_* = 804.98
Monoclinic, 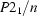

*a* = 11.497 (4) Å
*b* = 15.056 (5) Å
*c* = 15.749 (5) Å
β = 102.777 (15)°
*V* = 2658.5 (15) Å^3^
*Z* = 4
Mo *K*α radiation
μ = 4.57 mm^−1^
*T* = 293 K
0.21 × 0.20 × 0.18 mm

#### Data collection

Bruker SMART1000 CCD diffractometer
Absorption correction: multi-scan (*SADABS*; Sheldrick, 2003 ▶) *T*_min_ = 0.443, *T*_max_ = 0.489
19857 measured reflections
4653 independent reflections
4112 reflections with *I* > 2σ(*I*)
*R*_int_ = 0.028

#### Refinement

*R*[*F* ^2^ > 2σ(*F* ^2^)] = 0.034
*wR*(*F* ^2^) = 0.067
*S* = 1.08
4653 reflections
435 parameters
441 restraints
H-atom parameters constrained
Δρ_max_ = 1.37 e Å^−3^
Δρ_min_ = −1.18 e Å^−3^

### 

Data collection: *SMART* (Bruker, 2001 ▶); cell refinement: *SAINT-Plus* (Bruker, 2003 ▶); data reduction: *SAINT-Plus*; program(s) used to solve structure: *SHELXS97* (Sheldrick, 2008 ▶); program(s) used to refine structure: *SHELXL97* (Sheldrick, 2008 ▶); molecular graphics: *SHELXTL* (Sheldrick, 2008 ▶); software used to prepare material for publication: *SHELXL97*.

## Supplementary Material

Crystal structure: contains datablocks global, I. DOI: 10.1107/S1600536810048245/zs2074sup1.cif

Structure factors: contains datablocks I. DOI: 10.1107/S1600536810048245/zs2074Isup2.hkl

Additional supplementary materials:  crystallographic information; 3D view; checkCIF report

## Figures and Tables

**Fig. 1.**
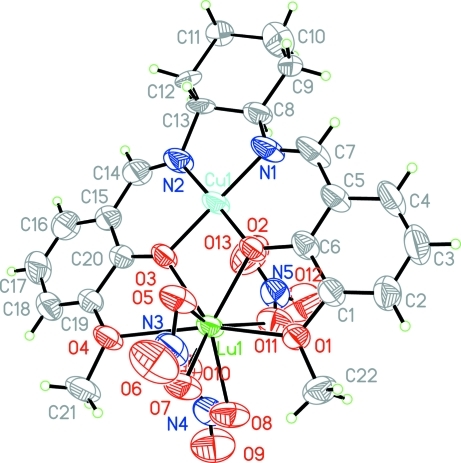
The molecular structure of the title compound, showing 50% probability displacement ellipsoids.

## supplementary crystallographic information

### Comment

In a continuation of the studies of salen-type lanthanide complexes (Mohamed *et al.* 2003, Aslantaş *et al.* 2007, Sun *et al.* 2009), we present here the crystal structure of the Cu^II^–Lu^III^ mixed-metal complex prepared using the ligand *N*,*N*'-bis(2-oxo-3-methoxybenzylidene)-1,2-diaminocyclohexane (H_2_L), [CuLu(C_22_H_24_N_2_O_4_)(NO_3_)_3_], the title compound (I) (Fig. 1).  The irregular LuO_9_ coordination sphere of (I) comprises two phenolate and two methoxy O donors from the imine-phenolate ligand molecule and five O-donors from the three nitrate anions, two bidentate and one monodentate. The Lu—Cu distance is 3.2336 (10) Å, but is not nitrate-bridged as found in analogous lanthanide complexes (Koner *et al.*, 2005; Sui *et al.*, 2006; Costes *et al.* 2008; Bao *et al.* 2010). Notably, the coordinated methanol molecule found in the Cu^II^–Eu^III^ complex (Bao *et al.*, 2010) is absent in the structure of (I), which is consistent with the familiar lanthanide contraction concept. The Lu—O bond distance range in (I) is 2.230 (3)–2.621 (4) Å. The Cu^II^ ion is tetra-coordinated by two imine N atoms and two bridging phenolate O atoms from the ligand molecule and is slightly distorted square-planar[Cu—N, 1.903 (4), 1.912 (4) Å; Cu—O, 1.897 (3), 1.906 (3) Å]. All atoms of the cyclohexane ring of the ligand molecule are 50% disordered.

### Experimental

To a 2:3 MeOH/MeCN solution (35 ml) of [(H_2_L)Lu(NO_3_)_3_] (0.2415 g, 0.3 mmol) was added an aqueous solution (10 ml) of Cu(OAc)_2_ . H_2_O (0.0597 g, 0.3 mmol) at ambient temperature. The color of the solution changed to red immediately and after stirring for 5 h, the solution was filtered to remove suspended particles. Red single crystals of the title compound suitable for single crystal X-ray determination were obtained by slow diffusion of diethylether into the filtrate over a period of one week. Elemental analysis: Calc. for C_22_H_24_N_5_O_13_CuLu: C, 32.82; H, 3.01; N, 8.70%, Found: C, 32.80; H, 3.09; N, 8.74%.

### Refinement

H atoms bound to C atoms were placed in calculated positions and treated as riding on their parent atoms, with C—H = 0.93 Å (aromatic C), C—H = 0.97 Å (methylene C), and with *U*_iso_(H) = 1.2*U*eq(C) or C—H = 0.96 Å (methyl C) and with *U*_iso_(H) = 1.5*U*eq(C). The C8—C13 atoms of the cyclohexane ring were refined as disordered over two sites with equivalent occupancy [site occupancy factor 0.50 (1)].

### Crystal data

**Table:**

[CuLu(C_22_H_24_N_2_O_4_)(NO_3_)_3_]	*F*(000) = 1580
*M**_r_* = 804.98	*D*_x_ = 2.011 Mg m^−^^3^
Monoclinic, *P*2_1_/*n*	Mo *K*α radiation, λ = 0.71073 Å
Hall symbol: -P 2yn	Cell parameters from 4112 reflections
*a* = 11.497 (4) Å	θ = 3.0–25.0°
*b* = 15.056 (5) Å	µ = 4.57 mm^−^^1^
*c* = 15.749 (5) Å	*T* = 293 K
β = 102.777 (15)°	Block, red
*V* = 2658.5 (15) Å^3^	0.21 × 0.20 × 0.18 mm
*Z* = 4	

### Data collection

**Table:**

Bruker SMART1000 CCD diffractometer	4653 independent reflections
Radiation source: fine-focus sealed tube	4112 reflections with *I* > 2σ(*I*)
graphite	*R*_int_ = 0.028
ω scans	θ_max_ = 25.0°, θ_min_ = 3.0°
Absorption correction: multi-scan (*SADABS*; Sheldrick, 2003)	*h* = −13→13
*T*_min_ = 0.443, *T*_max_ = 0.489	*k* = −17→17
19857 measured reflections	*l* = −18→18

### Refinement

**Table:**

Refinement on *F*^2^	Primary atom site location: structure-invariant direct methods
Least-squares matrix: full	Secondary atom site location: difference Fourier map
*R*[*F*^2^ > 2σ(*F*^2^)] = 0.034	Hydrogen site location: inferred from neighbouring sites
*wR*(*F*^2^) = 0.067	H-atom parameters constrained
*S* = 1.08	*w* = 1/[σ^2^(*F*_o_^2^) + (0.0114*P*)^2^ + 9.3555*P*] where *P* = (*F*_o_^2^ + 2*F*_c_^2^)/3
4653 reflections	(Δ/σ)_max_ = 0.004
435 parameters	Δρ_max_ = 1.37 e Å^−^^3^
441 restraints	Δρ_min_ = −1.17 e Å^−^^3^

### Special details

**Table:**

Geometry. All e.s.d.'s (except the e.s.d. in the dihedral angle between two l.s. planes) are estimated using the full covariance matrix. The cell e.s.d.'s are taken into account individually in the estimation of e.s.d.'s in distances, angles and torsion angles; correlations between e.s.d.'s in cell parameters are only used when they are defined by crystal symmetry. An approximate (isotropic) treatment of cell e.s.d.'s is used for estimating e.s.d.'s involving l.s. planes.
Refinement. Refinement of *F*^2^ against ALL reflections. The weighted *R*-factor *wR* and goodness of fit *S* are based on *F*^2^, conventional *R*-factors *R* are based on *F*, with *F* set to zero for negative *F*^2^. The threshold expression of *F*^2^ > σ(*F*^2^) is used only for calculating *R*-factors(gt) *etc*. and is not relevant to the choice of reflections for refinement. *R*-factors based on *F*^2^ are statistically about twice as large as those based on *F*, and *R*- factors based on ALL data will be even larger.

### Fractional atomic coordinates and isotropic or equivalent isotropic displacement parameters (Å^2^)

**Table:**

	*x*	*y*	*z*	*U*_iso_*/*U*_eq_	Occ. (<1)
C1	0.1707 (5)	0.4007 (3)	0.3948 (3)	0.0521 (13)	
C2	0.2248 (6)	0.3315 (4)	0.4453 (4)	0.0705 (18)	
H1	0.2465	0.2801	0.4199	0.085*	
C3	0.2464 (7)	0.3401 (5)	0.5356 (4)	0.086 (2)	
H2	0.2809	0.2933	0.5709	0.103*	
C4	0.2176 (6)	0.4163 (4)	0.5725 (4)	0.0738 (19)	
H3	0.2332	0.4207	0.6329	0.089*	
C5	0.1650 (5)	0.4885 (4)	0.5221 (3)	0.0548 (14)	
C6	0.1385 (5)	0.4784 (3)	0.4308 (3)	0.0503 (13)	
C7	0.1453 (6)	0.5704 (4)	0.5642 (3)	0.0690 (19)	
H4	0.1582	0.5696	0.6246	0.083*	
C8'	0.0550 (15)	0.7291 (9)	0.5641 (8)	0.062 (3)	0.50
H8'A	−0.0320	0.7335	0.5458	0.074*	0.50
C9'	0.106 (2)	0.7362 (15)	0.6651 (14)	0.082 (5)	0.50
H9'A	0.0598	0.6972	0.6943	0.098*	0.50
H9'B	0.1874	0.7154	0.6784	0.098*	0.50
C10'	0.1024 (16)	0.8222 (10)	0.6984 (9)	0.076 (4)	0.50
H10A	0.1333	0.8227	0.7609	0.091*	0.50
H10B	0.0214	0.8449	0.6859	0.091*	0.50
C11'	0.1827 (19)	0.8792 (15)	0.6517 (12)	0.110 (6)	0.50
H11A	0.1932	0.9381	0.6773	0.132*	0.50
H11B	0.2606	0.8518	0.6587	0.132*	0.50
C12'	0.125 (2)	0.8857 (15)	0.5580 (13)	0.119 (8)	0.50
H12A	0.0428	0.9047	0.5512	0.143*	0.50
H12B	0.1663	0.9292	0.5300	0.143*	0.50
C13'	0.1288 (14)	0.8034 (9)	0.5195 (8)	0.055 (3)	0.50
H13A	0.2119	0.7844	0.5265	0.066*	0.50
C8	0.1362 (15)	0.7261 (8)	0.5719 (7)	0.060 (3)	0.50
H8A	0.2159	0.7408	0.5634	0.072*	0.50
C10	0.1822 (16)	0.8067 (11)	0.7051 (10)	0.087 (4)	0.50
H10C	0.2668	0.8161	0.7095	0.104*	0.50
H10D	0.1695	0.8057	0.7639	0.104*	0.50
C11	0.1112 (16)	0.8930 (9)	0.6553 (7)	0.071 (4)	0.50
H11C	0.0363	0.8996	0.6734	0.110*	0.50
H11D	0.1584	0.9459	0.6735	0.110*	0.50
C13	0.0601 (13)	0.8020 (9)	0.5240 (7)	0.050 (3)	0.50
H13B	−0.0216	0.7897	0.5295	0.060*	0.50
C9	0.1550 (14)	0.7263 (15)	0.6706 (15)	0.052 (4)	0.50
H9A	0.0829	0.7054	0.6865	0.098*	0.50
H9B	0.2188	0.6853	0.6949	0.098*	0.50
C12	0.0855 (14)	0.8866 (11)	0.5565 (9)	0.048 (4)	0.50
H12C	0.0185	0.9250	0.5324	0.140*	0.50
H12D	0.1542	0.9088	0.5367	0.140*	0.50
C14	0.0372 (6)	0.8460 (4)	0.3697 (4)	0.0646 (16)	
H15	0.0373	0.9042	0.3895	0.077*	
C15	0.0098 (5)	0.8322 (3)	0.2756 (3)	0.0524 (13)	
C16	−0.0094 (6)	0.9073 (4)	0.2211 (4)	0.0676 (17)	
H16	−0.0069	0.9636	0.2459	0.081*	
C17	−0.0318 (6)	0.8992 (4)	0.1326 (4)	0.0731 (18)	
H17	−0.0424	0.9499	0.0980	0.088*	
C18	−0.0387 (6)	0.8158 (4)	0.0941 (4)	0.0623 (16)	
H18	−0.0550	0.8105	0.0338	0.075*	
C19	−0.0214 (5)	0.7422 (3)	0.1453 (3)	0.0490 (13)	
C20	0.0046 (5)	0.7484 (3)	0.2367 (3)	0.0463 (12)	
C21	−0.0363 (6)	0.6406 (4)	0.0246 (3)	0.0743 (18)	
H19	−0.1061	0.6700	−0.0079	0.112*	
H20	−0.0417	0.5781	0.0121	0.112*	
H21	0.0333	0.6644	0.0086	0.112*	
C22	0.1785 (6)	0.3256 (4)	0.2598 (4)	0.0677 (17)	
H22	0.2635	0.3188	0.2762	0.101*	
H24	0.1546	0.3340	0.1979	0.101*	
H23	0.1408	0.2733	0.2760	0.101*	
N1	0.1120 (5)	0.6435 (3)	0.5264 (3)	0.0609 (13)	
N2	0.0608 (5)	0.7850 (3)	0.4269 (3)	0.0619 (13)	
N3	−0.1133 (5)	0.4477 (3)	0.1669 (3)	0.0669 (14)	
N4	0.2518 (5)	0.5615 (4)	0.1211 (3)	0.0666 (14)	
N5	0.3397 (5)	0.6211 (4)	0.3520 (3)	0.0667 (13)	
O1	0.1432 (4)	0.4020 (2)	0.3038 (2)	0.0547 (10)	
O2	0.0835 (3)	0.5399 (2)	0.37526 (19)	0.0505 (9)	
O3	0.0256 (3)	0.6720 (2)	0.2799 (2)	0.0530 (9)	
O4	−0.0275 (4)	0.6544 (2)	0.1169 (2)	0.0568 (10)	
O5	−0.1043 (4)	0.4957 (3)	0.2343 (2)	0.0648 (11)	
O6	−0.2009 (5)	0.4029 (4)	0.1380 (3)	0.1028 (18)	
O7	−0.0232 (4)	0.4515 (3)	0.1322 (2)	0.0685 (11)	
O8	0.2100 (4)	0.4884 (3)	0.1397 (3)	0.0728 (12)	
O9	0.3266 (5)	0.5648 (4)	0.0781 (4)	0.1055 (18)	
O10	0.2120 (4)	0.6300 (3)	0.1509 (3)	0.0768 (13)	
O11	0.2908 (4)	0.5540 (3)	0.3091 (3)	0.0786 (13)	
O12	0.4475 (5)	0.6276 (4)	0.3708 (4)	0.113 (2)	
O13	0.2791 (5)	0.6785 (3)	0.3775 (4)	0.1015 (17)	
Lu1	0.09403 (2)	0.546272 (14)	0.234051 (13)	0.04774 (8)	
Cu1	0.07394 (6)	0.66185 (4)	0.40327 (4)	0.04949 (17)	

### Atomic displacement parameters (Å^2^)

**Table:**

	*U*^11^	*U*^22^	*U*^33^	*U*^12^	*U*^13^	*U*^23^
C1	0.069 (4)	0.046 (3)	0.039 (3)	−0.002 (3)	0.006 (3)	0.003 (2)
C2	0.094 (5)	0.051 (3)	0.060 (4)	0.011 (3)	0.005 (3)	0.008 (3)
C3	0.123 (6)	0.063 (4)	0.059 (4)	0.017 (4)	−0.008 (4)	0.018 (3)
C4	0.101 (5)	0.073 (4)	0.037 (3)	−0.001 (4)	−0.006 (3)	0.015 (3)
C5	0.075 (4)	0.054 (3)	0.032 (3)	−0.011 (3)	0.006 (3)	−0.001 (2)
C6	0.069 (4)	0.041 (3)	0.039 (3)	−0.008 (3)	0.008 (3)	0.003 (2)
C7	0.116 (5)	0.059 (4)	0.029 (3)	−0.027 (4)	0.008 (3)	−0.001 (2)
C8'	0.089 (7)	0.056 (6)	0.045 (5)	−0.013 (6)	0.026 (6)	−0.014 (5)
C9'	0.146 (15)	0.071 (10)	0.034 (7)	−0.027 (12)	0.031 (11)	−0.010 (7)
C10'	0.101 (8)	0.080 (7)	0.049 (6)	0.004 (7)	0.023 (6)	−0.021 (5)
C11'	0.123 (9)	0.103 (9)	0.091 (8)	−0.027 (8)	−0.002 (7)	−0.010 (7)
C12'	0.162 (16)	0.103 (12)	0.078 (11)	−0.066 (12)	−0.005 (11)	0.003 (10)
C13'	0.077 (7)	0.056 (6)	0.041 (5)	0.016 (6)	0.033 (6)	−0.007 (4)
C8	0.092 (7)	0.054 (6)	0.035 (5)	0.006 (6)	0.018 (6)	−0.008 (4)
C10	0.085 (7)	0.100 (8)	0.070 (7)	−0.005 (7)	0.006 (6)	0.009 (6)
C11	0.115 (11)	0.049 (7)	0.040 (6)	0.027 (7)	−0.002 (7)	−0.032 (5)
C13	0.061 (7)	0.061 (7)	0.031 (5)	0.007 (7)	0.015 (6)	−0.025 (5)
C9	0.061 (7)	0.060 (7)	0.036 (6)	0.019 (6)	0.010 (6)	−0.007 (5)
C12	0.055 (8)	0.048 (7)	0.047 (7)	0.015 (6)	0.023 (6)	−0.015 (5)
C14	0.098 (5)	0.039 (3)	0.054 (3)	0.010 (3)	0.013 (3)	−0.013 (3)
C15	0.063 (3)	0.046 (3)	0.048 (3)	0.006 (3)	0.012 (3)	−0.003 (2)
C16	0.093 (5)	0.041 (3)	0.068 (4)	0.010 (3)	0.017 (4)	−0.001 (3)
C17	0.101 (5)	0.052 (4)	0.063 (4)	0.015 (3)	0.011 (4)	0.021 (3)
C18	0.079 (4)	0.064 (4)	0.043 (3)	0.008 (3)	0.010 (3)	0.011 (3)
C19	0.061 (3)	0.049 (3)	0.035 (3)	0.005 (3)	0.008 (2)	−0.001 (2)
C20	0.057 (3)	0.045 (3)	0.037 (3)	0.006 (2)	0.010 (2)	−0.001 (2)
C21	0.108 (5)	0.080 (4)	0.033 (3)	0.001 (4)	0.014 (3)	−0.009 (3)
C22	0.095 (5)	0.045 (3)	0.068 (4)	0.008 (3)	0.031 (4)	−0.007 (3)
N1	0.102 (4)	0.050 (3)	0.030 (2)	−0.016 (3)	0.014 (2)	−0.0092 (19)
N2	0.104 (4)	0.043 (3)	0.038 (2)	0.004 (3)	0.012 (2)	−0.011 (2)
N3	0.091 (4)	0.059 (3)	0.046 (3)	−0.006 (3)	0.005 (3)	0.000 (2)
N4	0.081 (4)	0.068 (4)	0.050 (3)	−0.007 (3)	0.015 (3)	−0.002 (2)
N5	0.073 (4)	0.067 (4)	0.059 (3)	−0.001 (3)	0.012 (3)	0.006 (3)
O1	0.085 (3)	0.0361 (19)	0.0419 (19)	0.0078 (18)	0.0121 (19)	−0.0029 (15)
O2	0.083 (3)	0.0391 (19)	0.0280 (16)	0.0039 (18)	0.0084 (17)	−0.0006 (14)
O3	0.086 (3)	0.041 (2)	0.0275 (17)	0.0070 (18)	0.0029 (17)	−0.0018 (14)
O4	0.088 (3)	0.052 (2)	0.0285 (17)	0.000 (2)	0.0088 (18)	−0.0028 (15)
O5	0.089 (3)	0.067 (3)	0.040 (2)	−0.012 (2)	0.017 (2)	−0.0094 (18)
O6	0.111 (4)	0.110 (4)	0.079 (3)	−0.047 (3)	0.002 (3)	−0.023 (3)
O7	0.092 (3)	0.069 (3)	0.045 (2)	−0.010 (2)	0.016 (2)	−0.0190 (19)
O8	0.107 (4)	0.056 (3)	0.063 (3)	−0.018 (2)	0.035 (2)	−0.021 (2)
O9	0.106 (4)	0.124 (5)	0.106 (4)	−0.016 (3)	0.065 (4)	−0.002 (3)
O10	0.104 (4)	0.051 (3)	0.080 (3)	0.002 (2)	0.030 (3)	0.009 (2)
O11	0.081 (3)	0.090 (3)	0.060 (3)	−0.016 (3)	0.007 (2)	−0.021 (2)
O12	0.068 (3)	0.127 (5)	0.138 (5)	−0.010 (3)	0.008 (3)	−0.038 (4)
O13	0.094 (4)	0.065 (3)	0.153 (5)	−0.002 (3)	0.044 (4)	−0.002 (3)
Lu1	0.07542 (17)	0.03816 (12)	0.02938 (11)	0.00198 (11)	0.01104 (10)	−0.00581 (9)
Cu1	0.0809 (5)	0.0396 (3)	0.0265 (3)	−0.0002 (3)	0.0087 (3)	−0.0068 (2)

### Geometric parameters (Å, °)

**Table:**

C1—C2	1.374 (7)	C12—H12D	0.9700
C1—C6	1.386 (7)	C14—N2	1.273 (7)
C1—O1	1.397 (6)	C14—C15	1.460 (7)
C2—C3	1.395 (9)	C14—H15	0.9300
C2—H1	0.9300	C15—C20	1.398 (7)
C3—C4	1.360 (9)	C15—C16	1.407 (8)
C3—H2	0.9300	C16—C17	1.366 (8)
C4—C5	1.401 (8)	C16—H16	0.9300
C4—H3	0.9300	C17—C18	1.389 (8)
C5—C6	1.412 (7)	C17—H17	0.9300
C5—C7	1.440 (8)	C18—C19	1.360 (7)
C6—O2	1.333 (6)	C18—H18	0.9300
C7—N1	1.268 (7)	C19—O4	1.392 (6)
C7—H4	0.9300	C19—C20	1.408 (6)
C8'—C9'	1.57 (3)	C20—O3	1.330 (6)
C8'—N1	1.617 (15)	C21—O4	1.449 (6)
C8'—C13'	1.651 (19)	C21—H19	0.9600
C8'—H8'A	0.9800	C21—H20	0.9600
C9'—C10'	1.40 (3)	C21—H21	0.9600
C9'—H9'A	0.9700	C22—O1	1.446 (6)
C9'—H9'B	0.9700	C22—H22	0.9600
C10'—C11'	1.56 (3)	C22—H24	0.9600
C10'—H10A	0.9700	C22—H23	0.9600
C10'—H10B	0.9700	N1—Cu1	1.912 (4)
C11'—C12'	1.482 (17)	N2—Cu1	1.903 (4)
C11'—H11A	0.9700	N3—O6	1.214 (6)
C11'—H11B	0.9700	N3—O5	1.269 (6)
C12'—C13'	1.385 (17)	N3—O7	1.275 (6)
C12'—H12A	0.9700	N3—Lu1	2.809 (6)
C12'—H12B	0.9700	N4—O9	1.208 (7)
C13'—N2	1.520 (14)	N4—O10	1.260 (6)
C13'—H13A	0.9800	N4—O8	1.262 (6)
C8—N1	1.431 (13)	N4—Lu1	2.817 (6)
C8—C9	1.52 (3)	N5—O12	1.213 (7)
C8—C13	1.532 (18)	N5—O13	1.232 (7)
C8—H8A	0.9800	N5—O11	1.275 (6)
C10—C9	1.33 (3)	O1—Lu1	2.443 (3)
C10—C11	1.64 (2)	O2—Cu1	1.897 (3)
C10—H10C	0.9700	O2—Lu1	2.256 (3)
C10—H10D	0.9700	O3—Cu1	1.906 (3)
C11—C12	1.522 (14)	O3—Lu1	2.230 (3)
C11—H11C	0.9700	O4—Lu1	2.621 (4)
C11—H11D	0.9700	O5—Lu1	2.405 (4)
C13—C12	1.380 (18)	O7—Lu1	2.339 (4)
C13—N2	1.552 (11)	O8—Lu1	2.371 (4)
C13—H13B	0.9800	O10—Lu1	2.435 (4)
C9—H9A	0.9700	O11—Lu1	2.312 (4)
C9—H9B	0.9700	Lu1—Cu1	3.2336 (10)
C12—H12C	0.9700		
			
C2—C1—C6	122.1 (5)	H22—C22—H24	109.5
C2—C1—O1	125.1 (5)	O1—C22—H23	109.5
C6—C1—O1	112.8 (4)	H22—C22—H23	109.5
C1—C2—C3	118.4 (6)	H24—C22—H23	109.5
C1—C2—H1	120.8	C7—N1—C8	121.0 (6)
C3—C2—H1	120.8	C7—N1—C8'	128.6 (6)
C4—C3—C2	120.6 (6)	C7—N1—Cu1	125.3 (4)
C4—C3—H2	119.7	C8—N1—Cu1	110.9 (6)
C2—C3—H2	119.7	C8'—N1—Cu1	103.7 (5)
C3—C4—C5	121.9 (5)	C14—N2—C13'	122.1 (7)
C3—C4—H3	119.1	C14—N2—C13	122.1 (6)
C5—C4—H3	119.1	C14—N2—Cu1	125.3 (4)
C4—C5—C6	117.5 (5)	C13'—N2—Cu1	108.6 (6)
C4—C5—C7	119.7 (5)	C13—N2—Cu1	111.8 (6)
C6—C5—C7	122.7 (5)	O6—N3—O5	122.5 (6)
O2—C6—C1	116.7 (4)	O6—N3—O7	123.3 (5)
O2—C6—C5	123.9 (5)	O5—N3—O7	114.1 (5)
C1—C6—C5	119.4 (5)	O6—N3—Lu1	178.1 (5)
N1—C7—C5	126.1 (5)	O5—N3—Lu1	58.6 (3)
N1—C7—H4	117.0	O7—N3—Lu1	55.6 (3)
C5—C7—H4	117.0	O9—N4—O10	122.6 (6)
C9'—C8'—N1	109.6 (13)	O9—N4—O8	121.4 (6)
C9'—C8'—C13'	105.9 (13)	O10—N4—O8	116.0 (5)
N1—C8'—C13'	95.5 (9)	O9—N4—Lu1	174.5 (5)
C9'—C8'—H8'A	114.6	O10—N4—Lu1	59.6 (3)
N1—C8'—H8'A	114.6	O8—N4—Lu1	56.7 (3)
C13'—C8'—H8'A	114.6	O12—N5—O13	119.0 (6)
C10'—C9'—C8'	114.0 (17)	O12—N5—O11	120.0 (6)
C10'—C9'—H9'A	108.7	O13—N5—O11	120.9 (6)
C8'—C9'—H9'A	108.7	C1—O1—C22	117.1 (4)
C10'—C9'—H9'B	108.7	C1—O1—Lu1	116.8 (3)
C8'—C9'—H9'B	108.7	C22—O1—Lu1	123.6 (3)
H9'A—C9'—H9'B	107.6	C6—O2—Cu1	124.3 (3)
C9'—C10'—C11'	105.4 (15)	C6—O2—Lu1	123.7 (3)
C9'—C10'—H10A	110.7	Cu1—O2—Lu1	101.94 (14)
C11'—C10'—H10A	110.7	C20—O3—Cu1	124.7 (3)
C9'—C10'—H10B	110.7	C20—O3—Lu1	127.3 (3)
C11'—C10'—H10B	110.7	Cu1—O3—Lu1	102.56 (14)
H10A—C10'—H10B	108.8	C19—O4—C21	116.5 (4)
C12'—C11'—C10'	108.9 (17)	C19—O4—Lu1	112.4 (3)
C12'—C11'—H11A	109.9	C21—O4—Lu1	121.6 (3)
C10'—C11'—H11A	109.9	N3—O5—Lu1	94.7 (4)
C12'—C11'—H11B	109.9	N3—O7—Lu1	97.7 (3)
C10'—C11'—H11B	109.9	N4—O8—Lu1	96.9 (3)
H11A—C11'—H11B	108.3	N4—O10—Lu1	93.9 (3)
C13'—C12'—C11'	109.0 (18)	N5—O11—Lu1	125.2 (4)
C13'—C12'—H12A	109.9	O3—Lu1—O2	67.51 (12)
C11'—C12'—H12A	109.9	O3—Lu1—O11	99.63 (15)
C13'—C12'—H12B	109.9	O2—Lu1—O11	76.01 (15)
C11'—C12'—H12B	109.9	O3—Lu1—O7	123.28 (15)
H12A—C12'—H12B	108.3	O2—Lu1—O7	120.13 (14)
C12'—C13'—N2	121.6 (13)	O11—Lu1—O7	137.01 (16)
C12'—C13'—C8'	110.6 (15)	O3—Lu1—O8	143.33 (14)
N2—C13'—C8'	94.6 (10)	O2—Lu1—O8	139.68 (15)
C12'—C13'—H13A	109.6	O11—Lu1—O8	73.50 (16)
N2—C13'—H13A	109.6	O7—Lu1—O8	70.42 (16)
C8'—C13'—H13A	109.6	O3—Lu1—O5	82.32 (14)
N1—C8—C9	118.7 (13)	O2—Lu1—O5	74.19 (13)
N1—C8—C13	112.1 (10)	O11—Lu1—O5	146.74 (15)
C9—C8—C13	115.9 (11)	O7—Lu1—O5	53.48 (14)
N1—C8—H8A	102.3	O8—Lu1—O5	123.33 (14)
C9—C8—H8A	102.3	O3—Lu1—O10	90.71 (15)
C13—C8—H8A	102.3	O2—Lu1—O10	135.22 (14)
C9—C10—C11	118.5 (14)	O11—Lu1—O10	69.53 (16)
C9—C10—H10C	107.7	O7—Lu1—O10	104.60 (16)
C11—C10—H10C	107.7	O8—Lu1—O10	52.84 (15)
C9—C10—H10D	107.7	O5—Lu1—O10	143.69 (14)
C11—C10—H10D	107.7	O3—Lu1—O1	132.25 (12)
H10C—C10—H10D	107.1	O2—Lu1—O1	64.96 (11)
C12—C11—C10	113.7 (10)	O11—Lu1—O1	73.27 (15)
C12—C11—H11C	108.8	O7—Lu1—O1	78.86 (14)
C10—C11—H11C	108.8	O8—Lu1—O1	81.29 (14)
C12—C11—H11D	108.8	O5—Lu1—O1	80.95 (14)
C10—C11—H11D	108.8	O10—Lu1—O1	126.96 (15)
H11C—C11—H11D	107.7	O3—Lu1—O4	61.91 (11)
C12—C13—C8	117.1 (11)	O2—Lu1—O4	125.73 (12)
C12—C13—N2	118.1 (12)	O11—Lu1—O4	129.63 (15)
C8—C13—N2	103.6 (8)	O7—Lu1—O4	76.06 (13)
C12—C13—H13B	105.7	O8—Lu1—O4	94.21 (14)
C8—C13—H13B	105.7	O5—Lu1—O4	80.77 (13)
N2—C13—H13B	105.7	O10—Lu1—O4	64.75 (14)
C10—C9—C8	112.6 (18)	O1—Lu1—O4	154.56 (12)
C10—C9—H9A	109.1	O3—Lu1—N3	103.87 (15)
C8—C9—H9A	109.1	O2—Lu1—N3	97.06 (14)
C10—C9—H9B	109.1	O11—Lu1—N3	150.70 (16)
C8—C9—H9B	109.1	O7—Lu1—N3	26.73 (14)
H9A—C9—H9B	107.8	O8—Lu1—N3	96.78 (16)
C13—C12—C11	114.5 (14)	O5—Lu1—N3	26.76 (14)
C13—C12—H12C	108.6	O10—Lu1—N3	126.75 (15)
C11—C12—H12C	108.6	O1—Lu1—N3	78.00 (14)
C13—C12—H12D	108.6	O4—Lu1—N3	77.69 (13)
C11—C12—H12D	108.6	O3—Lu1—N4	117.21 (15)
H12C—C12—H12D	107.6	O2—Lu1—N4	143.95 (14)
N2—C14—C15	125.5 (5)	O11—Lu1—N4	67.95 (15)
N2—C14—H15	117.3	O7—Lu1—N4	88.31 (16)
C15—C14—H15	117.3	O8—Lu1—N4	26.40 (14)
C20—C15—C16	118.2 (5)	O5—Lu1—N4	140.35 (13)
C20—C15—C14	123.5 (5)	O10—Lu1—N4	26.51 (14)
C16—C15—C14	118.3 (5)	O1—Lu1—N4	103.84 (15)
C17—C16—C15	121.3 (5)	O4—Lu1—N4	79.76 (14)
C17—C16—H16	119.3	N3—Lu1—N4	114.62 (15)
C15—C16—H16	119.3	O3—Lu1—Cu1	35.12 (8)
C16—C17—C18	120.4 (5)	O2—Lu1—Cu1	35.02 (8)
C16—C17—H17	119.8	O11—Lu1—Cu1	78.00 (11)
C18—C17—H17	119.8	O7—Lu1—Cu1	139.31 (12)
C19—C18—C17	119.4 (5)	O8—Lu1—Cu1	150.09 (11)
C19—C18—H18	120.3	O5—Lu1—Cu1	85.91 (9)
C17—C18—H18	120.3	O10—Lu1—Cu1	108.32 (11)
C18—C19—O4	126.4 (5)	O1—Lu1—Cu1	99.18 (8)
C18—C19—C20	121.5 (5)	O4—Lu1—Cu1	97.00 (8)
O4—C19—C20	112.2 (4)	N3—Lu1—Cu1	112.67 (12)
O3—C20—C15	124.8 (4)	N4—Lu1—Cu1	130.54 (11)
O3—C20—C19	115.9 (4)	O2—Cu1—N2	177.78 (17)
C15—C20—C19	119.2 (5)	O2—Cu1—O3	81.92 (14)
O4—C21—H19	109.5	N2—Cu1—O3	95.94 (17)
O4—C21—H20	109.5	O2—Cu1—N1	94.81 (17)
H19—C21—H20	109.5	N2—Cu1—N1	87.29 (19)
O4—C21—H21	109.5	O3—Cu1—N1	174.90 (19)
H19—C21—H21	109.5	O2—Cu1—Lu1	43.04 (9)
H20—C21—H21	109.5	N2—Cu1—Lu1	135.12 (14)
O1—C22—H22	109.5	O3—Cu1—Lu1	42.32 (10)
O1—C22—H24	109.5	N1—Cu1—Lu1	135.74 (15)
